# Postnatal Feeding With a Fat Rich Diet Induces Precocious Puberty Independent of Body Weight, Body Fat, and Leptin Levels in Female Mice

**DOI:** 10.3389/fendo.2019.00758

**Published:** 2019-11-08

**Authors:** Rahim Ullah, Ali Raza, Naveed Rauf, Yi Shen, Yu-Dong Zhou, Junfen Fu

**Affiliations:** ^1^Department of Endocrinology, Children's Hospital, Zhejiang University School of Medicine, Hangzhou, China; ^2^Key Laboratory of Medical Neurobiology of the Ministry of Health of China, Department of Neurobiology, The Collaborative Innovation Center for Brain Science, Institute of Neuroscience, Zhejiang University School of Medicine, Hangzhou, China; ^3^National Clinical Research Center for Child Health, Chongqing, China

**Keywords:** postnatal overnutrition, body weight, body fat, leptin, precocious puberty

## Abstract

Puberty generally occurs when an individual has stored a sufficient amount of energy. Previous reports have shown that postnatal overfeeding, induced by a small litter size or maternal high fat diet (HFD) feeding during gestation and lactation increases body weight (BW), body fat, plasma leptin levels, and induces precocious puberty. The role of BW, body fat, and leptin in postnatal HFD-induced precocious puberty is poorly understood. In this study, we investigated if postnatal HFD feeding induces precocious puberty independent of BW, body fat, and leptin levels. Different litter sizes and different exposure time to HFD were used to produce HFD feeding pups with different BW and body fat. BW, body fat, and plasma hormones levels were checked at different time points to test their relation with HFD-induced precocious puberty. Our results showed that postnatal HFD feeding increases BW, body fat, adipocyte size, and induces precocious puberty. HFD-induced precocious puberty was independent of BW, body fat, and plasma leptin levels. Plasma gonadotrophin, estradiol, testosterone and insulin levels were comparable in most of the groups. Our results collectively suggest that postnatal HFD feeding induces precocious puberty independent of BW, body fat and plasma leptin levels. Our results also suggest that HFD feeding acts as a stimulator for puberty onset but further studies are needed to understand how it induces precocious puberty.

## Introduction

Puberty is an important developmental stage of the life span when an individual achieves somatic and reproductive maturity (1–3). Central neurotransmitters, neurohormones, and environmental cues integrate on the hypothalamic-pituitary-gonadal axis (HPG-axis) and regulate reproduction and puberty onset (2, 4, 5). The HPG-axis is activated by GnRH neurons during puberty, which results in gonadal maturation and function (2, 6). GnRH is regulated by various afferent regulatory signals including leptin, insulin, proopiomelanocortin (POMC), agouti-related peptide (AGRP), and kisspeptin (7–10).

Nutritional status including overnutrition, undernutrition, and overall metabolic alterations during perinatal stages affect puberty onset. Postnatal overnutrition and HFD feeding induce earlier puberty onset (11–13); however, undernutrition delays puberty onset (13). Postnatal HFD feeding induces obesity (4, 11, 13) and obesity is associated with increased circulating leptin levels (14). There has been great interest in the association between HFD, body fat, leptin signaling, and puberty. Mice with a deleted obese gene (ob), are incapable of producing leptin and cannot pass through puberty unless treated with exogenous leptin (15). Similarly, delayed puberty due to undernutrition in mice and rats has been shown to be reversible, unless too severe, via leptin administration (16, 17). Likewise, leptin administration has advanced puberty onset in normally fed female mice (18). Leptin is produced in adipocytes and circulates in accordance with the fat stored in the body (19). The body fat hypothesis, proposed by Frisch, suggests that “a minimum level of stored, easily mobilized energy is necessary for ovulation and menstrual cycles in the human female” (20). The modern hypothesis suggests that puberty begins when body fat and circulating leptin levels reach optimum levels (15).

This picture is very complex in rodents and it should also be noted that it is not so straightforward in humans either. One study reported that circulating leptin levels increase during peripubertal stages in female rats (21); however, another study found no such alterations (22). Similarly, Ahima et al. (23) found that leptin levels increase during the second postnatal week. However, there is no increase thereafter even during the time when the first estrus cycle begins (23). Consistent with later findings, Bronson found that puberty is not associated with either body fat and plasma leptin as they found that both decrease toward puberty onset (24).

Postnatal HFD feeding increases BW and body fat and induces precocious puberty (4, 11, 25). Little is known about whether postnatal HFD feeding induces precocious puberty by increasing BW, body fat, and plasma leptin. The present study was designed to address the hypothesis that postnatal HFD feeding induces precocious puberty independent of BW, body fat, and circulating leptin levels. To test this hypothesis, we changed the litter size and feeding paradigms to dams and pups and successfully developed HFD feeding pups with different BW, body fat, and leptin levels.

## Materials and Methods

### Animals

C57BL/6J female mice were used in the current study. Mice were housed in the animal facility of Zhejiang University under the standard conditions for air, humidity, and temperature on a 12 h light:12 h dark cycle (lights on at 6:00 A.M.). The animals had free access to food and water. At the age of 12 weeks, male and female mice were paired for breeding. Female pups were used in this experiment. This study was approved by the National Institutes of Health Guidelines for the Care and Use of Laboratory Animals and Animal Advisory Committee at Zhejiang University.

### Experimental Design

**Figure 1:**
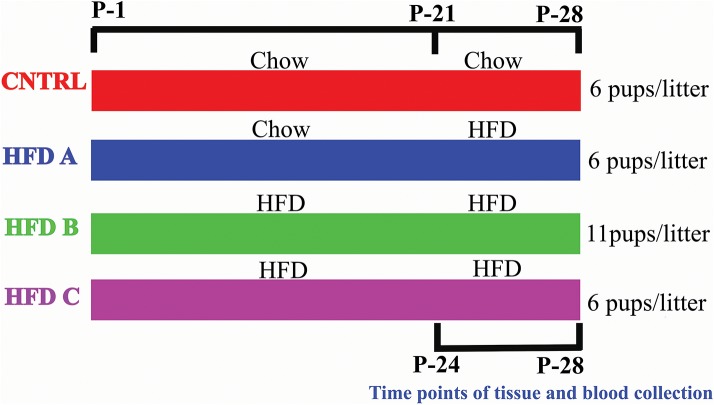
Graphical representation of the feeding paradigm of dams and pups.

To investigate whether postnatal HFD feeding induces precocious puberty independent of BW, body fat, and plasma leptin, it was necessory to produce mouse pups that would feed on a HFD but have a normal body weight, at least before puberty onset. Litter size affects body weight. A small litter size induces obesity, and a large litter size results in undernutrition and reduced body weight (26, 27). The dams of all groups were fed a control diet (10% fat) [Research Diets Inc., New Brunswick, New Jersey, USA] before and during pregnancy. The dams and pups of the control group were fed a control diet after delivery and post-weaning, respectively. The pups numbered 6 in each litter and this group was named as CNTRL. Three HFD groups were designed. The dams of one group were fed with a control diet during lactation, however the pups were fed on a HFD (60% fat) [HFD, Research Diets Inc., New Brunswick, New Jersey, USA] post-weaning. The pups numbered 6 in each litter and this group was labeled HFD A. The dams and pups of the second group were fed a HFD during lactation and post-weaning, respectively; however, the number of pups were 11 per litter. This group was named HFD B. The feeding paradigm of the third HFD group was similar to HFD B, however the number of pups per litter was 6. This group was named HFD C. Both male and female pups were present in each litter. There were four dams and 13 pups per group in the presented study. CNTRL and HFD C were taken as control groups. The large litter size and post-weaning HFD feeding produced pups with normal BW and precocious puberty. The litter sizes were similar to previous studies (27–29). The birth day was considered as postnatal day 1 (P-1). Experimental design is shown in Figure 1.

### Body Weight, Body Fat, and Observation of Vaginal Canalization (VC)

BW and body fat were measured as an index of obesity. Vaginal canalization (VC) has been used as a marker of puberty in rodents (30–33). Pups were regularly checked for weight gain and VC from weaning (at P-21) until puberty onset in all of the groups. Given that obese female pups reached puberty at approximately P-28. P-24 and P-28 were selected to represent the time points before and after puberty onset.

### Tissue Preparation for Histomorphological Analysis

The animals were killed and epigonadal fat pads were dissected at P-24. Epigonadal fats were fixed in 4% PFA (Sigma-Aldrich) for 48 h. After fixation, tissue samples were subjected to ascending grades of alcohol for dehydration. The tissue samples were then embedded in paraffin and blocks were prepared for microtomy. Seven micrometer thick sections were cut by microtome (CM30503, Leica) and stained with hematoxylin and eosin (H&E) [Sigma-Aldrich]. The sections were then subjected to descending grades of alcohol for rehydration. Stained sections were viewed under a light microscope (BX51; Olympus, Tokyo, Japan) and pictures were taken. Mean adipocyte surface area was determined in H&E stained sections. The cell sizes of all (~100–120) adipocytes within each picture from at least four animals per group were measured by using image J, version 1.40 (NIH, Bethesda, MD, USA).

### Blood Hormones Assays

Blood samples were collected from the heart at P-24 and P-28 and centrifuged at 3,000 g for 15 min. Blood plasma was collected and stored at −80°C until the experiment. Leptin ELISA kits (CSB-E0671m, CUSABIO, USA) with a sensitivity of 2.5 IU/ml and a co-efficient of variation (CV) of <15% and (CK-E42895, Gold Wheat, China) with 0.1 ng/ml sensitivity and 15% coefficient of variation were used for assessing plasma leptin levels. Plasma LH levels were measured by using commercial ELISA kits (CSB-E0671m, CUSABIO, USA) with 0.5 IU/ml sensitivity and <15% CV and (CK-E43318, Gold Wheat, China) with 0.1 mIU/ml sensitivity and 15% CV. Plasma FSH levels were measured by using commercial ELISA kits (CSB-E0671m, CUSABIO, USA) with 2.5 IU/ml sensitivity, 15% CV and (CK-E43291, Gold Wheat, China) with 1 mIU/ml sensitivity and 15% CV. Plasma E2 levels were measured by using a commercial ELISA kit (CK-E43302, Gold Wheat, China). The sensitivity of the E2 assay was 1 pmol/ml and the CV was 15%. Plasma Insulin levels were measured by using a commercial ELISA kit (CK-E20353, Gold Wheat, China). The sensitivity of the Insulin assay was 0.1 mIU/L and the CV was 15%. Plasma Testosterone levels were measured by using a commercial ELISA kit (CK-E20375, Gold Wheat, China). The sensitivity of the Testosterone assay was 1 pG/ml and the CV was 15%. The assays were performed according to the manufacturer's protocol.

### Statistical Analysis

Data are presented as mean ± SEM. Student's unpaired *t*-test and one-way ONVA were used for different analyses. *p* < 0.05 was considered as significant.

## Results

### Postnatal Feeding With a HFD Increases BW, Body Fat, and Induces Precocious Puberty

**Figure 2:**
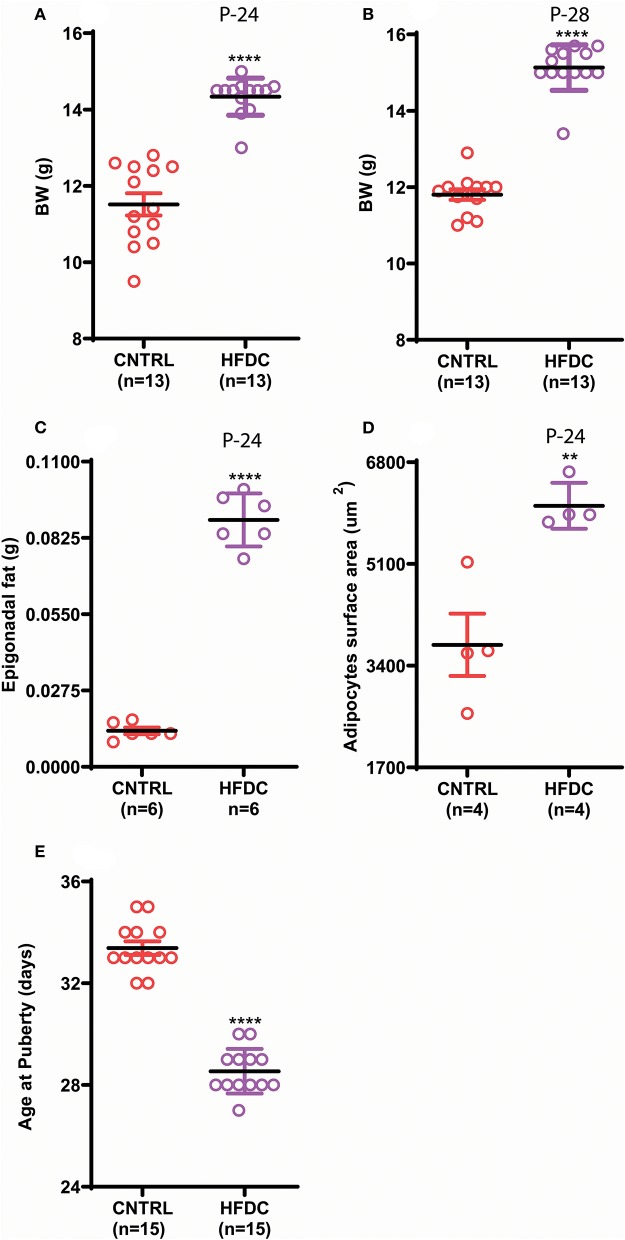
Postnatal feeding with HFD induces obesity and precocious puberty. **(A)** Body weight at P-24, **(B)** body weight at P-28, **(C)** weight of Epigonadal white edipose tissues at P-24, **(D)** surface area of adipocytes at P-24, **(E)** age of puberty onset. Un-paired *t*-test was used for analyses and data are presented as mean ± SEM. **p < 0.01, ****p < 0.0001.

Postnatal overnutrition induced by raising pups in a small litter size increases body weight (26). To check whether postnatal HFD feeding induces obesity before puberty onset in female C57BL/6J mouse pups, we provided a HFD to dams after delivery and to pups post-weaning and checked body weight, body fat and day of VC. Postnatal HFD feeding significantly increased body weight (Figures 2A,B). Interestingly, HFD feeding showed an increasing tendency to gain BW from P-24 to P-28; however, it was almost constant in CNTRL pups. Furthermore, the epigonadal fat deposition (Figure 2C) and adipocyte size (Figure 2D and Supplementary Figure 1) were significantly increased in HFD feeding pups compared to the CNTRL group. Furthermore, HFD feeding pups showed earlier VC compared to CNTRL pups (Figure 2E). Of note, here HFD and control mean HFD C and CNTRL, respectively. Our results demonstrate that postnatal HFD feeding induces obesity and precocious puberty in C57BL/6J mouse pups.

### Postnatal Feeding With HFD Induces Precocious Puberty Independent of Body Weight

To investigate whether postnatal HFD feeding induces precocious puberty independent of body weight, we changed the litter size and feeding paradigm and produced HFD feeding mouse pups with a normal body weight (explained in the experimental design and Figure 1).

**Figure 3:**
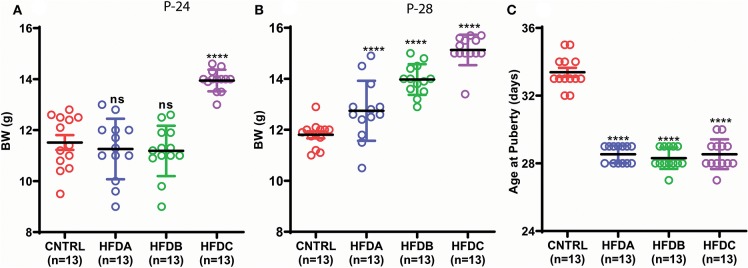
Postnatal HFD feeding induces precocious puberty independent of body weight. **(A)** Body weight at P-24, **(B)** body weight at P-28, **(C)** age at puberty onset. A one way ANOVA was used for analyses and data are presented as mean ± SEM. *p < 0.05, ****p < 0.0001.

The BW of HFD A and HFD B were simialar to CNTRL and significanly lower than HFD C at P-24 (Figure 3A); however, HFD A and HFD B were significantly heavier than CNTRL but significanly lighter than HFD C at P-28 (Figure 3B). Regardless of different BW before and at (nearly) puberty, all the HFD feeding pups showed earlier VC compared to CNTRL (Figure 3C).

### Postnatal Feeding With HFD Induces Precocious Puberty Independent of Body Fat and Plasma Leptin Levels

**Figure 4:**
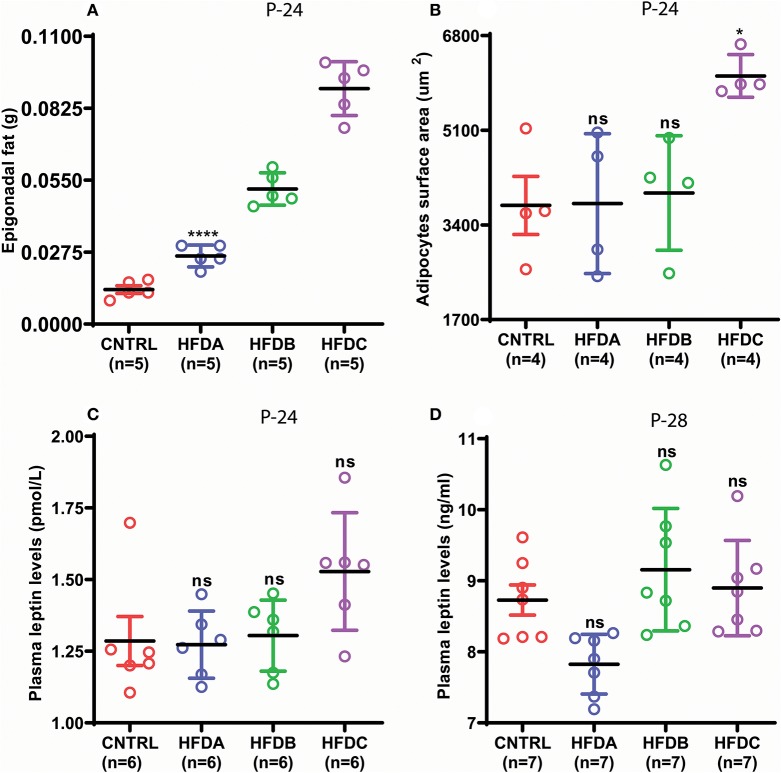
Postnatal HFD feeding induces precocious puberty independent of body fat. **(A)** weight of epigonadal white adipose tissues at P-24, **(B)** surface area of adipocytes at P-24, **(C)** plasma leptin levels at P-24, **(D)** plasma leptin levels at P-28. A one way ANOVA was used for analyses and data are presented as mean ± SEM. *p < 0.05,****p < 0.0001.

Next, we investigated whether postnatal HFD feeding induces precocious puberty independent of body fat and plasma leptin levels. HFD feeding pups in all the three groups showed significantly increased fat deposition compared to the CNTRL group (Figure 4A). However, fat deposition in HFD groups was also significantly different from one another. Interestingly, adipocyte size in CNTRL, HFD A, and HFD B was comparable (Figure 4B and Supplementary Figure 1); however, adipocyte size in HFD C pups was significantly larger compared to CNTRL, HFD A, and HFD B groups at P-24. A previous study found that puberty onset increases body fat (24). P-28 is the time point of puberty onset in HFD pups. Therefore, to avoid confusion regarding whether fat deposition at puberty (P-28) is the result of HFD feeding or puberty onset, we only checked body fat before puberty onset (P-24). Leptin is a trophic hormone, secreted by adipocytes in response to the energy status of the body and acts primarily in the hypothalamus to regulate the metabolism and reproduction (34, 35). Therefore, we subsequently checked whether postnatal feeding has any effect on plasma leptin levels. Plasma leptin in all the HFD groups was comparable with the CNTRL group (Figures 4C,D). Interestingly, regardless of differences in epigonadal fat deposition, adipocyte size, and leptin levels, all the HFD feeding pups (HFD A, HFD B, and HFD C) showed early puberty compared to CNTRL (Figure 3C). Our results demonstrate that HFD feeding induces precocious puberty independent of body fat and plasma leptin levels.

### Effects of Postnatal HFD Feeding on Reproductive Hormones

**Figure 5:**
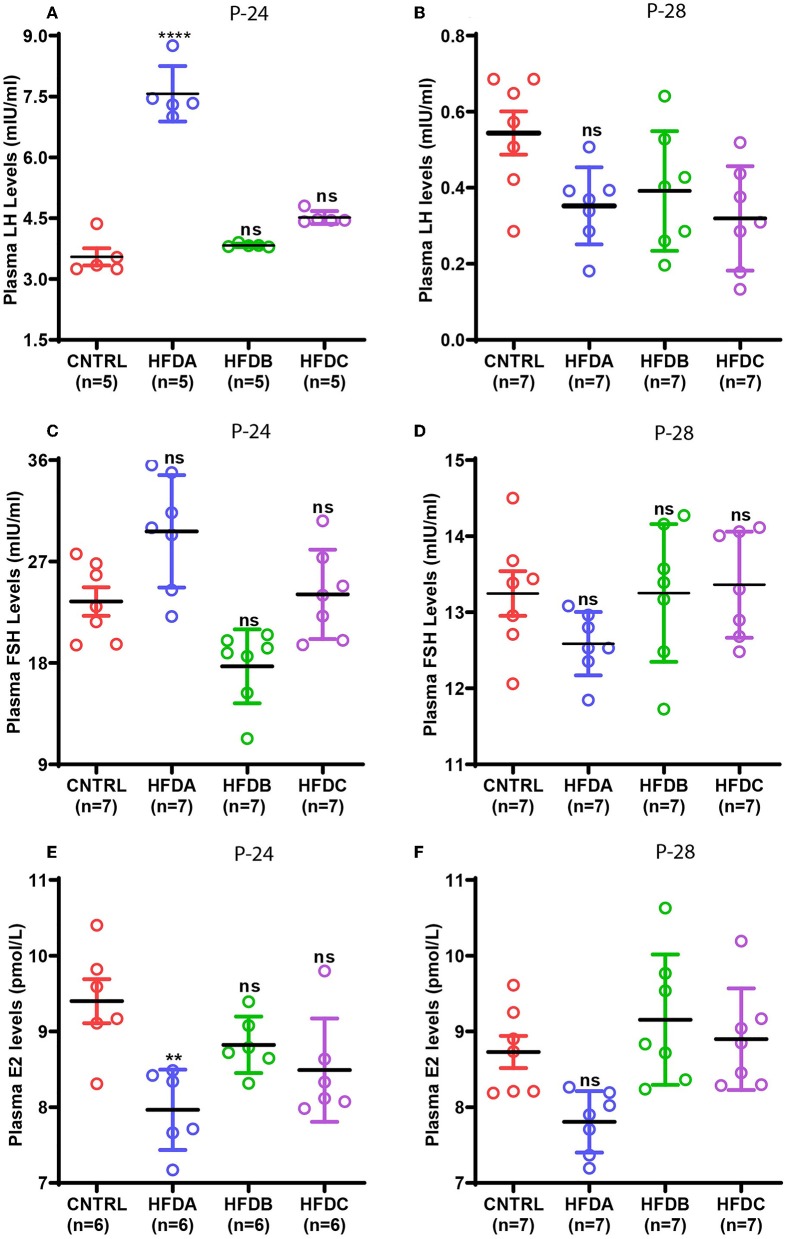
Effects of postnatal HFD feeding on plasma levels of sex hormones. **(A)** Plasma LH at P-24, **(B)** plasma LH at P-28, **(C)** plasma FSH at P-24, **(D)** plasma FSH at P-28, **(E)** plasma E2 at P-24, **(F)** plasma E2 at P-28. A one way ANOVA was used for analyses and data are presented as mean ± SEM. *p < 0.05, **p < 0.01, ****p < 0.0001.

Gonadotropins and estradiol secreted by the pituitary gland and ovaries, respectively, are the principal sex hormones stimulated by upstream hypothalamic neuropeptides that regulate reproduction in animals (7). To reveal the effects of postnatal HFD feeding on reproductive hormones, the plasma levels of LH, FSH, and E2 were checked at P-24 (before puberty) and at P-28 (at puberty onset). Compared to the CNTRL, HFD B, and HFD C groups, significantly elevated levels of LH were found in the HFD A group at P-24 (Figure 5A); however, LH levels in HFD groups were significantly lower compared to CNTRL at P-28 (Figure 5B). Similarly, FSH levels at P-24 were significantly elevated in HFD A compared to the rest of the groups (Figure 5C); however, it was comparable in all the groups at P-28 (Figure 5D). Interestingly, E2 levels were significantly lower in the HFD A group compared to the rest of the groups, both at P-24 and P-28 (Figures 5E,F). Our results suggest that short-term exposure to HFD may stimulate gonadotropin levels. However, long-term exposure may have inhibitory effects on sex hormones.

Furthermore, a previous study found that post-weaning HFD feeding mediates early puberty through insulin and androgen (36). To investigate the mechanism of HFD induced early puberty in our animal model, we checked plasma insulin and testosterone levels at P-24 and P-28. Paradoxically, we found no rise in either insulin or testosterone levels, both at P-24 and P-28 (Supplementary Figures 2, 3).

## Discussion

In the current study, we tried to investigate whether postnatal HFD feeding induces precocious puberty independent of BW, body fat and leptin levels. Admittedly, due to the nature and design of our experiment, we did not investigate some interesting variables, including some neuropeptide expression and neuronal circuitry, which limits the mechanistic insight of our study; however, our experiments/analyses successfully proved our hypothesis. We found that HFD feeding to dams during lactation and to pups post-weaning triggers BW gain, body fat deposition and induces adipocyte hyperplasia that ultimately result in obesity. HFD feeding also induced precocious puberty. Our results are consistent with our previous findings (4) and other studies demonstrating that over-nutrition and HFD feeding increase BW, epigonadal fat deposition and induce adipocyte hyperplasia and precocious puberty (37–42). Various factors including high energy proficiency of HFD (43), HFD-induced hypothalamic inflammation (44), consumption of long chain fatty acid (LFAs) rich milk by pups (45) and postnatal HFD-induced hyperphagy in pups may contribute to the observed obesity in our experiment. During energy surplus conditions, adipocytes undergo hypertrophy to accommodate more fatty acids and keep blood glucose and fatty acids below toxic levels (46) and therefore we observed adipocyte hyperplasia in obese mouse pups.

Furthermore, we found that postnatal HFD feeding induces precocious puberty independent of BW, body fat and plasma leptin levels. Our results can be supported by previous studies. Bronson (24) reported that body fat strongly decreases toward puberty and increases only after puberty onset in mice. Similarly, female mice that were exposed to male mice showed precocious puberty with lower BW and body fat compared to isolated females (24). Likewise, HFD feeding induces earlier estrus at lower BW and body fat compared to control rat pups (47). Furthermore, bisphenol A treatment induces precocious puberty in female mice at lower BW compared to a control group (48). Consistent with our results, regardless of reducing pubertal BW, kisspeptin, and RF9 (an agonist of kisspeptin) administrations induce precocious puberty (49). These results collectively suggest that BW and body fat are not critical for puberty onset when other factors directly or indirectly activate puberty onset. Our data suggest that HFD-induced obesity and precocious puberty are independent functions of postnatal HFD feeding but a HFD has the potential to induce precocious puberty and obesity; therefore, we see both at the same time during HFD feeding.

To reveal the mechanism of HFD-induced precocious puberty, we checked plasma leptin levels. Interestingly, postnatal HFD feeding induces precocious puberty but has no effect on leptin levels. Our results are consistent with a previous study where HFD feeding after weaning induced precocious puberty but did not affect plasma leptin during puberty in rats (13). Bronson (24) reported that leptin levels steeply decrease toward puberty and increase after puberty onset. Furthermore, female mice exposed to male mice showed precocious puberty but had lower plasma leptin levels compared to isolated and grouped female mice (24). This study suggests that puberty by itself increases body fat and plasma leptin. Similarly, a previous study found that post-weaning HFD feeding mediates precocious puberty through insulin and androgen signaling (36). However, we found no rise in either insulin or androgen levels before or at puberty onset in HFD groups. Our results are consistent with a study in humans showing no association of insulin with menarche (50). Taken together, our study suggests that postnatal HFD feeding affects puberty onset separately from its effects on leptin and insulin levels.

Next, we investigated whether postnatal HFD feeding has any effects on reproductive hormones including LH, FSH, and E2 in our animal model. Elevated LH and FSH levels were found in the HFD A group before puberty onset; however, it was reduced at puberty onset in all the HFD groups. Consistent with our findings, a previous study reported that HFD feeding has no effect on circulating LH and FSH levels during puberty in rats (13). Similarly, exposure of pubertal mice to HFD for 10 weeks reduced LH levels compared to a control group. However, FSH levels were not affected (51). Prolonged exposure to HFD represses reproduction (51, 52). Based on the above evidence, it can be speculated that postnatal HFD exposure initially increases gonadotropin levels that trigger puberty onset. However, continuous exposure suppresses it and therefore, compared to CNTRL, we see elevated levels of LH and FSH in the HFD A group before puberty onset but reduced or comparable LH and FSH levels in all the HFD groups during puberty. Furthermore, E2 levels were comparable in all the groups at P-24 and P-28. Our results are consistent with the previous study where postnatal HFD feeding did not affect E2 levels during puberty in mice (25). However, we do not know why E2 is reduced in the HFD A group at P-24 and P-28.

In summary, our results suggest that postnatal HFD feeding induces precocious puberty through mechanisms other than BW, body fat, and plasma leptin levels. It seems that postnatal HFD feeding may affect some hypothalamic neurocircuitry involved in the metabolism and reproduction that mediate HFD-induced precocious puberty. However, further studies are required to prove it.

## Data Availability Statement

All datasets generated for this study are included in the article/Supplementary Material.

## Ethics Statement

The animal study was reviewed and approved by Animal Care and Use Committee of Zhejiang University.

## Author Contributions

RU, Y-DZ, and JF designed experiments. RU, NR, and AR performed the experiments. RU analyzed the data and wrote the paper. Y-DZ, JF, and YS edited the manuscript.

### Conflict of Interest

The authors declare that the research was conducted in the absence of any commercial or financial relationships that could be construed as a potential conflict of interest.

## Supplementary Material

Supplementary Figure 1 Micrograph representing adipocyte size.

Supplementary Figure 2 Effects of postnatal HFD feeding on plasma levels of Insuline. **(A)** Plasma Insulin at P-24, **(B)** plasma Insulin at P-28. Un-paired *t*-test was used for analyses and data are presented as mean ± SEM. ^*^*p* < 0.05, ^**^*p* < 0.01, ^***^*p* < 0.001.

Supplementary Figure 3 Effects of postnatal HFD feeding on plasma levels of Testosterone. **(A)** Plasma Testoterone at P-24, **(B)** plasma Testoterone at P-28. Un-paired *t*-test was used for analyses and data are presented as mean ± SEM.
