# Supplementary figures and images for: Postnatal Feeding With a Fat Rich Diet Induces Precocious Puberty Independent of Body Weight, Body Fat, and Leptin Levels in Female Mice
Source: Front Endocrinol (Lausanne). 2019 Nov 8;10:758. doi: 10.3389/fendo.2019.00758 (PMC6856215; doi:10.3389/fendo.2019.00758)

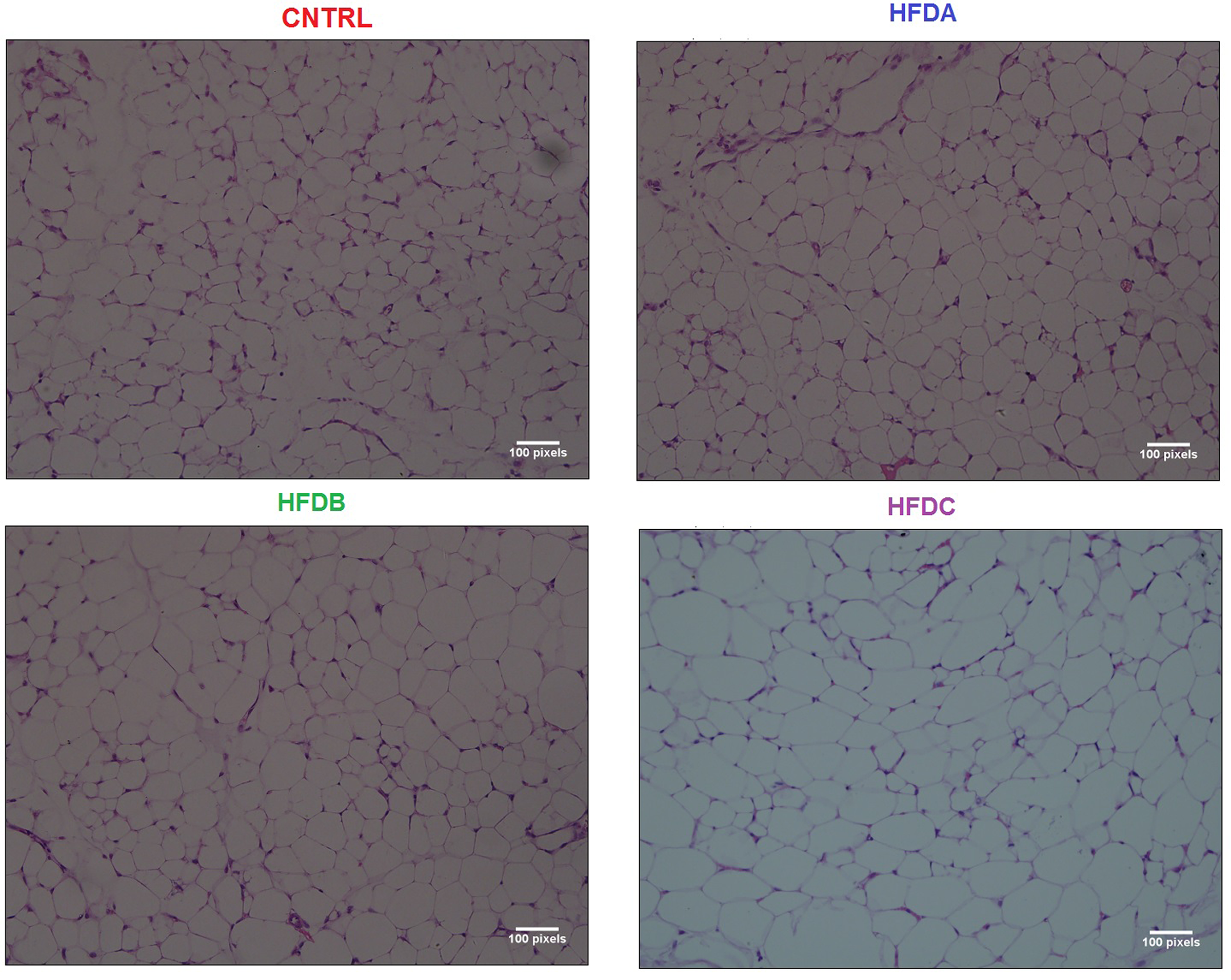

Supplement: Supplementary Figure 1 — Micrograph representing adipocyte size. [file Image_1.TIF]

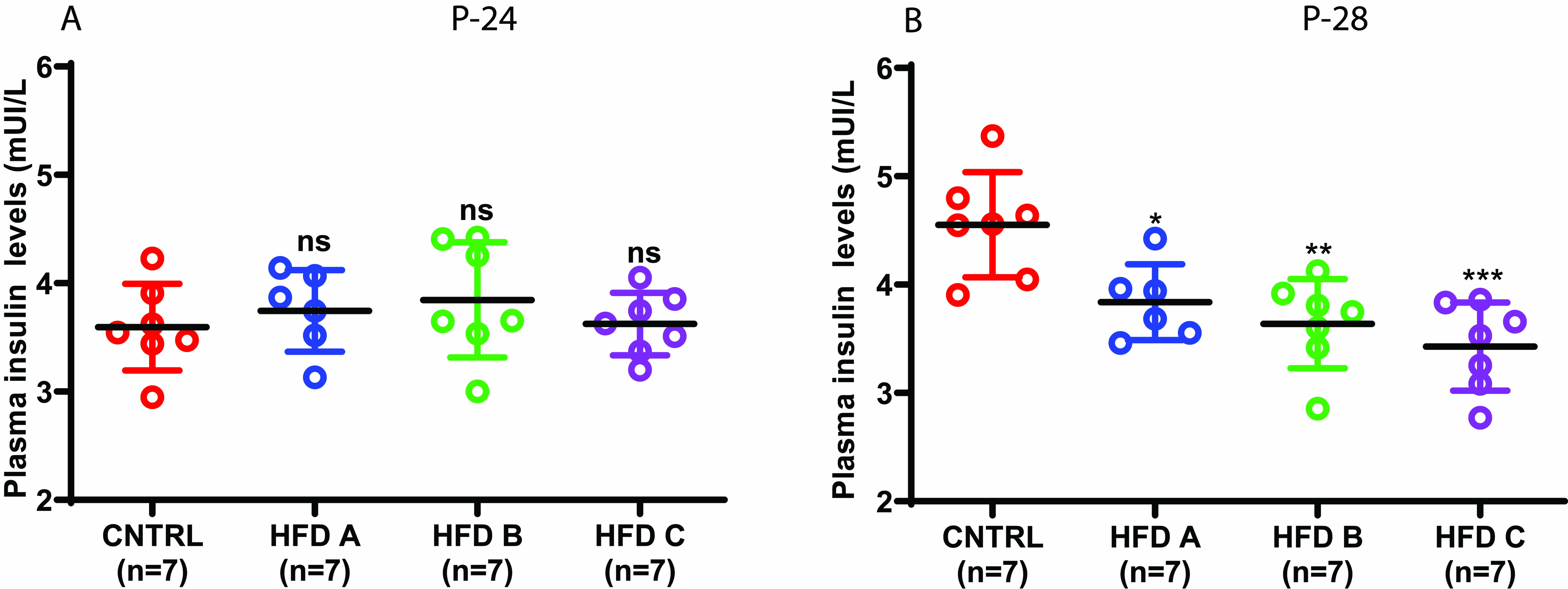

Supplement: Supplementary Figure 2 — Effects of postnatal HFD feeding on plasma levels of Insuline. (A) Plasma Insulin at P-24, (B) plasma Insulin at P-28. Un-paired t-test was used for analyses and data are presented as mean ± SEM. *p < 0.05, **p < 0.01, ***p < 0.001. [file Image_2.jpg]

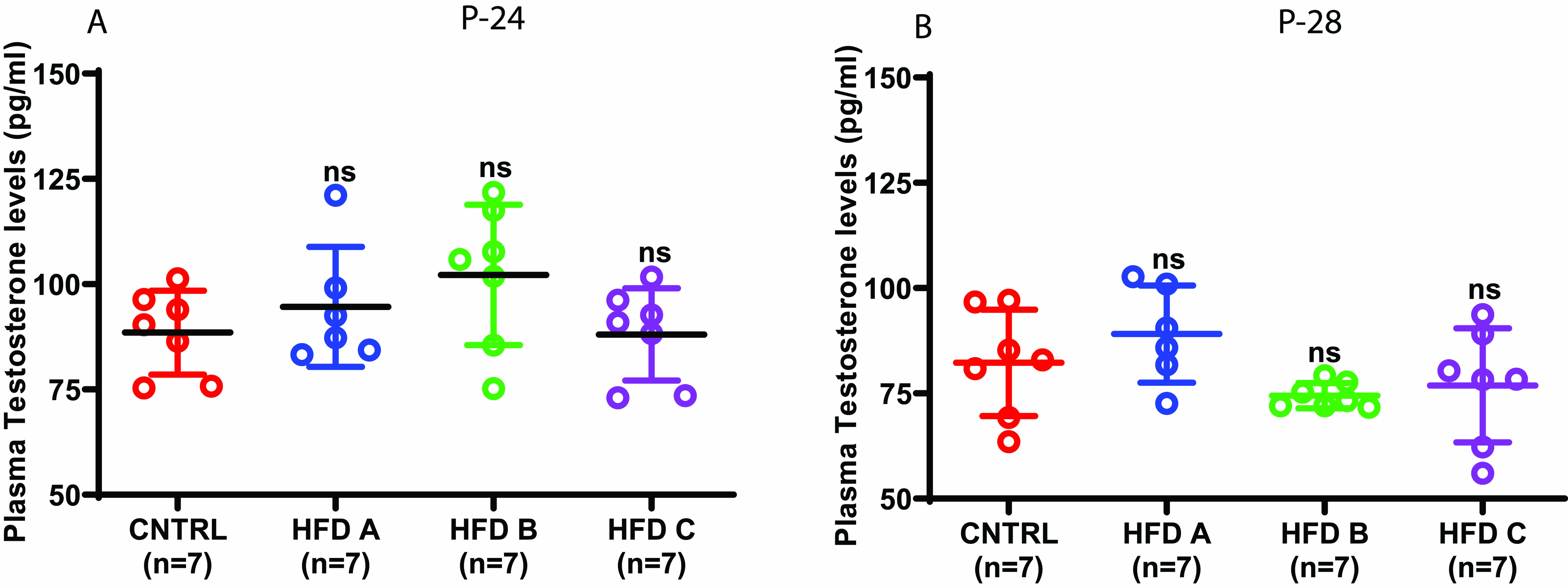

Supplement: Supplementary Figure 3 — Effects of postnatal HFD feeding on plasma levels of Testosterone. (A) Plasma Testoterone at P-24, (B) plasma Testoterone at P-28. Un-paired t-test was used for analyses and data are presented as mean ± SEM. [file Image_3.jpg]
